# Expressive communication skills: impact on content engagement and personal branding in Jordan

**DOI:** 10.3389/fsoc.2026.1848223

**Published:** 2026-08-06

**Authors:** Islam Habis Mohammad Hatamleh

**Affiliations:** Department of Media and Communication Technology, Faculty of Media, Jadara University, Irbid, Jordan

**Keywords:** content engagement, expressive communication skills, Jordan, personal branding, social media, university students

## Abstract

This study examines how expressive communication skills influence content engagement and personal branding among university students in Jordan. A quantitative, cross-sectional survey was administered online to 400 Jordanian university students. The measurement model comprised three constructs—expressive communication skills, content engagement, and personal branding—measured using established multi-item scales and analyzed with Partial Least Squares Structural Equation Modeling (PLS-SEM). The results showed that expressive communication skills had a significant positive effect on content engagement (β = 0.214, *t* = 2.900, *p* = 0.004) and personal branding (β = 0.543, *t* = 11.962, *p* < 0.001). Expressive communication skills explained 4.6% of the variance in content engagement and 29.5% of the variance in personal branding. These findings indicate that expressive communication skills contribute to online interaction but play a substantially stronger role in personal branding. The study concludes that expressive communication skills are an effective component of digital communication and identity construction among university students in Jordan, while content engagement is also shaped by additional content-, platform-, and audience-related factors.

## Introduction

The proliferation of online platforms has reshaped communication practices, particularly in contexts such as Jordan, where social media functions as a major tool for communication, cultural expression, and social interaction (Amer, 2024). Within this digital environment, expressive communication skills are increasingly important for shaping content engagement and personal branding (Abdallah et al., 2024). Social media platforms in Jordan are not merely leisure spaces; they also contribute to identity formation, social approval, and the negotiation of traditional values with global online trends among young people (Migdadi and Abukhadair, 2025). This relationship between self-expression and the digital environment requires closer examination of how Jordanians use communication styles to construct online identities and connect with different audiences (Rababah and Banikalef, 2018).

In particular, the strategic use of communication styles, including authenticity, humor, and visual storytelling, plays a significant role in audience engagement and digital identity formation among members of Generation Z (Nurrachmah, 2025). The need to understand these dynamics is further supported by the high level of internet penetration in Jordan and the widespread use of social media among young people (Galdón-Salvador et al., 2024).

Digital platforms have opened new avenues for self-expression and identity formation, especially for women who are seeking personal and professional development (Amiri and Ghourdou, 2024; Tartory, 2019). In this context, expressive communication skills go beyond the technical aspects and involve the use of language creatively, the emotional appeal of the language, and multimodal presentation strategies that influence the perception and interaction with the content by the audience.

Moreover, the phenomenon of using English in addition to Arabic in the online communication of younger Jordanians is a distinct sociolinguistic phenomenon that affects the communication pattern and the audience reception (Aburqayiq et al., 2025). This linguistic duality can make it difficult to build effective expressive communication, as it can influence perception of personal branding in the various online communities. This is important when considering the role these skills play in Jordan's digital landscape, where communal values are prevalent and social media consumption is high (Omeish et al., 2025). Especially Generation Z users use expressive communication to react to public opinion and create a recognizable personal brand (Alsharairi et al., 2025).

Previous studies emphasize the role of linguistic and semiotic factors in successful online self-presentation and make comparisons with other research on self-presentation strategies in other Arab settings (Muyidi, 2025). The content creators, especially Arab women vloggers, tend to negotiate their gendered identities and apply multimodal discourse to form coherent self-representations and advance alternative narratives that challenge hegemonic perceptions (Mitrić, 2022). Determining the linguistic and visual markers that organize performative self-representation gives an idea of how narrators create coherence and authenticity in their online identity (Lomborg et al., 2022). Moreover, the ability to switch between Arabic and English also greatly influences the perception of the audience and participation in personal branding projects (Amer, 2024), which confirms the strategic character of expressive communication.

The reasons why freelance professionals use social media to build their personal brand also show the connection between expressive communication and the formation of professional identity (Barley et al., 2017). The intentional use of linguistic features, such as code-mixing of Arabic and English, may serve as a proactive expressive communication tool to improve the results of engagement and branding (Al-Smadi, 2017; Sabila et al., 2025; Vanyushina et al., 2021). In the same vein, the use of local cultural stories and aesthetics creates a feeling of community and belongingness among the Jordanian viewers, which enhances brand loyalty (Mohamed, 2025). Nevertheless, authenticity is the key element; the fine line between relatability and perceived inauthenticity has a strong influence on the effectiveness of personal branding (Delport and Mulder, 2023).

Moreover, the use of multiple languages helps to connect with the wider international audience and maintain the local relevance (Limatius, 2023). These practices help to create hybrid identity that appeals to both traditional and modern influences (Çupi and Alimerko, 2024; Pujiati et al., 2025). Linguistic fluidity, and especially code-switching, is not only communicative convenience but also a fundamental process in the creation of inclusive digital identities in heterogeneous virtual communities (Ahmad, 2025; Zebua et al., 2025). This linguistic skill is particularly acute in the context of professional branding, when people are able to use the platform features strategically to create a favorable image and gain opportunities (Mou and Faruk, 2024). Finally, linguistic options, multimodal content creation, and identity formation play a significant role in authenticity, social capital, and interaction with the audience on such platforms as Tik Tok and Instagram (Hussain et al., 2025; Putikadyanto et al., 2021).

Although the literature on digital identity, multilingual practices, and online interaction is expanding, empirical studies that systematically examine the direct relationship between expressive communication skills, content engagement, and personal branding in Jordan remain limited. Most previous studies examine isolated practices, such as authenticity, code-switching, influencer style, or multimodal presentation, rather than testing expressive communication skills as an integrated predictor of both engagement and personal branding. Evidence from Jordan is especially limited, leaving unclear whether the same expressive capacities have comparable effects on immediate audience interaction and longer-term identity construction.

Accordingly, this study addresses the gap by integrating self-presentation, digital identity, and relational communication perspectives in a parsimonious model. Its novelty lies in testing, within an underexamined Jordanian context, whether expressive communication skills differentially predict content engagement and personal branding. This design extends existing research by distinguishing between an audience-response outcome and an identity-building outcome rather than treating digital self-expression as a single, undifferentiated consequence. The study is guided by the following research questions:

**RQ1:** What is the impact of expressive communication skills on content engagement?

**RQ2:** What is the impact of expressive communication skills on personal branding?

## Literature review

### Expressive communication skills

Expressive communication Expressive communication skills refer to a wide spectrum of verbal and non-verbal messages that are used strategically to create meaning, emotion, and intent, and thus influence the perception and involvement of the audience. These skills are essential in the digital world as they help content creators to create their online identity, which affects how they are viewed by their audience and develop their brand (Ezzat, 2020). This entails a deliberate use of language, such as tactical code-switching between Arabic and English, and multimodal components to create an impressive digital identity (Azzahro and Silvhiany, 2025). Such a complex negotiation of identity and audience interaction is becoming more common in a wide range of digital platforms, which is a strategic integration of linguistic codes (Wulandari et al., 2025). As an example, the strategic application of code-switching between a local language and a global one, such as English, can help not only identify with the culture but also gain wider popularity in the digital environment (Alhadidi et al., 2025; Nani, 2024). This linguistic flexibility, comprising such elements as slang and emojis, is not merely a form of communication, but also an essential process by which individuals construct and perform various elements of their identities within online communities (Puspitasari et al., 2024). This identity construction, conscious or unconscious, is a discursive practice that reveals itself in online communication, namely in the commentaries and other interactive web resources (Li, 2024).

### Content engagement

Content engagement can be understood as the complex interactions and relationships between users and digital content, which include not only the viewership but also the active engagement, resonance, and interest. This interaction is often facilitated by language and semiotic tools that shape user attitudes and desires in real-time online spaces (Kang et al., 2023). In particular, the strategic placement of the reader and the development of interpersonal meaning based on lexicogrammatically options play a vital role in promoting the involvement in online communication, especially in cross-cultural situations (Alshehri and Imran, 2025). Specifically, the intentional integration of various semiotic tools, such as speech, gestures, and images, is essential to attract online audiences to digital media (Xia and Hafner, 2021). Moreover, the successful use of linguistic styles by influencers is a major factor in triggering digital consumer behavior on such platforms as YouTube (Munaro et al., 2024). This is also complicated by the private linguistic practices of online communication, where people are strategically controlling their self-presentation and identity formation using language (Lee, 2019). This is an active process of building digital identity that is usually influenced by the linguistic repertoires and community standards that are dominant in different online platforms (András and Balázs, 2025; Matrood, 2025).

### Personal branding

Personal branding in online environments is the deliberate construction and communication of a recognizable public image through content and interaction, with potential consequences for an individual's reputation and opportunities. Performative actions such as posting, commenting, and liking communicate preferences and identity and contribute to a mediated public persona (Aporbo, 2023).

In addition, personal branding is a deliberate process through which individuals develop and communicate a distinctive identity to achieve personal, social, or professional goals. Khedher (2014) describes personal branding as a planned process involving three main stages: establishing personal brand identity, developing brand positioning through communication and behavior, and evaluating brand image. This view supports the present study because expressive communication skills help students communicate their values, abilities, personality, and identity in ways that can strengthen their online personal brand.

It is a crucial performative characteristic of identity work that is crucial to credibility and authenticity that is vital to draw and keep an audience in competitive digital spaces (Tzanne and Sifianou, 2019). Moreover, personal branding can be interpreted as a form of consumer interaction, where influencers are the personal brands and their followers are the consumers, which form relationships that transcend content consumption (Zhang, 2025). This dynamic is often motivated by the ability of the influencer to effectively combine multimodal and multilingual practices, which raises the degree of authenticity and ultimately results in commercial success (Włodarczyk et al., 2023). The strategic execution of these integrated communication strategies can help individuals not only to be capable of delivering their own value proposition, but also to be capable of being able to shine in the saturated digital markets. It is a deliberate creation of a social image, which can be achieved by a deliberate blend of professional authority and communicative forms that are easy to associate with, allowing influencers to build intimacy and credibility with their followers (Torjesen, 2024).

### Theoretical foundation: self-presentation, social media identity, and relational communication

This study is based on the theories of self-presentation, digital identity, and relational communication. Goffman's theory of self-presentation suggests that people control the impression that others have of them through their verbal and non-verbal actions. Communication is thus not only the sharing of information, but also a performance in which people try to project a desired image. This perspective is pertinent because the ability to express oneself clearly, confidently and strategically in digital environments is a function of expressive communication skills (Goffman, 1949; Burgoon and Hale, 1984). This is even more important in the social media environment where it is actively produced by the users themselves in their profiles, posts, pictures, comments, and interactions. Ellison (2013), states that the social media platforms offer opportunities for selective self-presentation, construction of identity and audience management. Likewise, Kaplan and Haenlein (2010) describe social media as being based on Web 2.0 technologies and enabling the creation and sharing of user-generated content. Thus, these platforms provide opportunities for individuals to express themselves, interact with an audience and create a digital identity.

Relational communication theory also backs up the importance of expressive communication skills in digital communication. Relational messages, according to Burgoon and Hale (1984), convey significant social implications of intimacy, similarity, trust, control, and involvement. For this study, students who are more expressive in their communication may be better able to express trustworthiness, authenticity, confidence and social closeness in their online content. These relational meanings can enhance the audience interaction and help build personal brand.

Therefore, expressive communication skills can be understood as a key mechanism through which students manage online impressions, communicate relational meaning, and construct personal brands on social media.

## Hypothesis development

### Expressive communication skills and content engagement

Expressive communication skills are expected to positively influence content engagement because audience interaction partly depends on how clearly, emotionally, and relationally content is communicated. Social media engagement is reflected in actions such as viewing, reacting, commenting, sharing, and producing user responses (Barger et al., 2016). Clear, authentic, and expressive messages are more likely to be perceived as relatable and to invite interaction.

Prior research suggests that authenticity, multimodal communication, linguistic style, and the attractiveness of message delivery can increase online audience interaction (Abdallah et al., 2024; Galdón-Salvador et al., 2024; Nurrachmah, 2025; Xia and Hafner, 2021). Expressive communication may therefore turn ideas and emotions into content that is easier to understand, more engaging, and more likely to be watched, liked, commented on, or shared.

Relational communication theory also supports this expectation. Burgoon and Hale (1984) argue that messages convey relational meanings, including affection, involvement, similarity, and social orientation. On social media, these meanings may encourage users to respond through likes, comments, and shares. Accordingly, students with stronger expressive communication skills are expected to achieve higher content engagement in the Jordanian context. H1: Expressive Communication Skills significantly and positively influence Content Engagement among university students in Jordan.

### Expressive communication skills and personal branding

The ability to express communication skills is also expected to enhance personal branding, as it will help people to project themselves in a clear, authentic and consistent manner on digital platforms. As mentioned in previous studies cited in this paper, self-presentation, authenticity, and construction of digital identity are significantly influenced by communication practices (Amer, 2024; Labrecque et al., 2011; Mitrić, 2022; Tewatia and Majumdar, 2022), which is a key part of personal branding. Thus, students who are more expressive communicators are more likely to create a unique and authentic personal brand in Jordan's social media landscape. Accordingly, the following hypothesis is proposed: **H2: Expressive Communication Skills significantly and positively influence Personal Branding among university students in Jordan**.

## Methodology

The study used a quantitative research design to investigate the effect of expressive communication skills on content engagement and personal branding from the perspective of university students in Jordan. The use of quantitative methods in communication and social media research is widespread because they enable researchers to measure the relationships between variables and statistically test theoretical assumptions (Creswell and Creswell, 2017). The study design was a cross-sectional survey, a method that allows data to be collected from many participants at a single point in time and allows for the efficient analysis of relationships between variables (Bryman, 2016). SmartPLS was used to test the proposed hypotheses. A bootstrapping procedure with 5,000 resamples was performed to evaluate the significance of structural model relationships. Standard errors, *t*-values, and *p*-values were produced for the hypothesized paths in this procedure. A two tailed significance test was used at 5% level, and the results were judged by the suggested critical value of t = 1.96. The method used was to find out whether the relationship between expressive communication skills, content engagement, and personal branding was statistically significant or unsignificant.

## Population and sample

The target population consisted of the university students in Jordan as they are among the most active users of social media platforms and digital communication tools. University students are a suitable target group for studying expressive communication in the digital environment, as they are often engaged in content creation, communication and digital self-presentation. The sampling method used was convenience sampling. The convenience sampling method is widely used in social science studies when the researcher chooses the participants according to their availability and readiness to take part (Etikan et al., 2016). A sample of 400 university students was used to gather data, which is deemed to be large enough to be used in structural equation modeling. Sarstedt et al. (2021) state that the bigger the sample size, the higher the statistical power and stability of the Partial Least Squares Structural Equation Modeling (PLS-SEM version 4.1.1.8) results. Since participation depended on accessibility and willingness, the sample should not be interpreted as statistically representative of all Jordanian university students.

## Data collection procedure

The data were gathered through an online questionnaire, which is considered to be one of the most effective ways of collecting data among digitally active groups of people, including university students (Dillman et al., 2014). The questionnaire was sent using different online platforms, such as social media and messaging apps that are popular among students. The survey was conducted on a voluntary basis. The purpose of the study was explained to the respondents and they were assured that their answers would be anonymous and confidential. Online surveys are especially appropriate when it comes to the studies concerning digital communication behaviors since they enable the respondents to answer the questions within the same digital space where the behaviors take place (Sarstedt et al., 2021). Therefore, data collection started only after ethical approval had been granted. The questionnaire was distributed during the period from **03-01-2026 to 06-05-2026** through different online platforms, including social media and messaging applications commonly used by university students in Jordan. A total of 500 students were invited to participate in the survey. Of these, 430 responses were received. After excluding 30 incomplete responses, 400 valid questionnaires were retained for analysis, resulting in a valid response rate of 80.0%.

## Measurement instrument

The questionnaire was divided into two parts. The initial part was the demographic profile of the respondents as indicated in Table 1, which included the variables of gender, age, type of university, level of study, academic year, field of study, residence, and social media usage characteristics. The second part was used to measure the main study constructs, which are Expressive Communication Skills, Content Engagement, and Personal Branding as illustrated in Table 2. These constructs were measured with multi-item scales based on previous research and were measured on a five-point Likert scale between 1 (strongly disagree) and 5 (strongly agree). The outer loadings and the reliability/validity indicators of the constructs are also reported in Table 2, which proves the sufficiency of the measurement model.

**Table 1:** Demographic profile of respondents (*N* = 400).

No.	Variable	Category	Frequency	Percentage (%)
1	Gender	Male	188	47.0
		Female	212	53.0
2	Age	18–20 years	126	31.5
		21–23 years	174	43.5
		24–26 years	72	18.0
		27 years and above	28	7.0
3	University type	Public	238	59.5
		Private	162	40.5
4	Level of study	Bachelor's	332	83.0
		Master's	52	13.0
		PhD	16	4.0
5	Academic year	First year	74	18.5
		Second year	96	24.0
		Third year	104	26.0
		Fourth year	86	21.5
		Fifth year and above	40	10.0
6	Field of study	Business	78	19.5
		Humanities	64	16.0
		Engineering	82	20.5
		Information Technology	70	17.5
		Medical Sciences	58	14.5
		Other	48	12.0
7	Place of residence	North Jordan	96	24.0
		Central Jordan	224	56.0
		South Jordan	80	20.0
8	Daily social media usage	Less than 1 h	24	6.0
		1–3 h	122	30.5
		4–6 h	156	39.0
		More than 6 h	98	24.5
9	Most frequently used platform	Instagram	118	29.5
		TikTok	104	26.0
		Facebook	62	15.5
		X	24	6.0
		Snapchat	54	13.5
		YouTube	28	7.0
		Other	10	2.5
10	Main purpose of social media use	Entertainment	138	34.5
		Education	70	17.5
		Communication	82	20.5
		Personal Branding	46	11.5
		Content Sharing	48	12.0
		Other	16	4.0
11	Content creation experience	Yes	244	61.0
		No	156	39.0
12	Posting frequency	Daily	84	21.0
		Weekly	126	31.5
		Monthly	74	18.5
		Rarely	82	20.5
		Never	34	8.5

**Table 2:** Measurement items, outer loadings, and reliability/validity assessment.

Construct	Item code	Measurement item	Source	Outer loading	Cronbach's alpha	rho_A	Composite reliability (rho_C)	AVE
Expressive Communication Skills	ECS1	I wait for others to finish their words before I take the turn to speak.	Abdallah et al. (2024)	0.832	0.892	0.895	0.925	0.755
	ECS2	I can express my thoughts clearly whenever I want.	Abdallah et al. (2024)	0.869	0.892	0.895	0.925	0.755
	ECS3	I can understand the emotions underlying what others are saying.	Abdallah et al. (2024)	0.875	0.892	0.895	0.925	0.755
	ECS4	I can easily start a conversation with other people.	Abdallah et al. (2024)	0.898	0.892	0.895	0.925	0.755
Content Engagement	CE1	When social media influencers upload photos and video clips of brands on social media, I watch them.	Mir and Salo (2024)	0.820	0.907	0.912	0.931	0.730
	CE2	I click the like button when the SMI shares video clips of brands on social media.	Mir and Salo (2024)	0.865	0.907	0.912	0.931	0.730
	CE3	I click the like button when SMI shares pictures of brands on social media.	Mir and Salo (2024)	0.873	0.907	0.912	0.931	0.730
	CE4	I engage in commenting on the SMI-generated brand-related photos and video clips.	Mir and Salo (2024)	0.848	0.907	0.912	0.931	0.730
	CE5	I share brand-related SMI-generated pictures and video clips with my friends and followers on social media.	Mir and Salo (2024)	0.865	0.907	0.912	0.931	0.730
Personal Branding	PB1	I consciously manage how I present myself on social media.	Labrecque et al. (2011)	0.867	0.911	0.913	0.934	0.739
	PB2	I try to create a consistent personal image through my online posts.	Labrecque et al. (2011)	0.890	0.911	0.913	0.934	0.739
	PB3	I use social media to highlight my skills and achievements.	Labrecque et al. (2011)	0.885	0.911	0.913	0.934	0.739
	PB4	I carefully select the content I share to reflect my personality.	Labrecque et al. (2011)	0.867	0.911	0.913	0.934	0.739
	PB5	I want others to recognize my personal identity through my online presence.	Labrecque et al. (2011)	0.783	0.911	0.913	0.934	0.739

ECS, Expressive Communication Skills; CE, Content Engagement; PB, Personal Branding; AVE, Average Variance Extracted.

Table 1 presents the demographic profile of the respondents (*N* = 400). The demographic profile of the respondents that participated in the study is provided in Table 1 (*N* = 400). The sample was fairly balanced in terms of gender, with 212 females (53.0%) and 188 males (47.0) which means that there was a slight dominance of females. In terms of age, the highest percentage of respondents was 2,123 years (43.5%), then 1,820 years (31.5%), and the remaining 2,427 years and above (18.0%). This implies that the sample was primarily comprised of young adults, which is in line with the target population of university students.

In terms of university type, many of the respondents were studying in public universities (59.5%), with 40.5% studying in private universities. Regarding study, most of the respondents were undertaking a bachelor degree (83.0%), with 13.0% pursuing a master's degree and 4.0% pursuing a PhD degree. In the same way, the distribution by academic year revealed that the highest percentage of respondents was in the third year (26.0%), second year (24.0%), and fourth year (21.5%), with fewer respondents in the first year (18.5%), and fifth year or above (10.0%).

In addition, the respondents had different academic backgrounds. Engineering (20.5%), Business (19.5%), Information Technology (17.5%), Humanities (16.0%), and Medical Sciences (14.5%) had the highest number with 20.5, 19.5, 17.5, 16.0 and 14.5 percent respectively, and 12.0 percent of the total were other specializations. Regarding place of residence, over fifty percent of the respondents resided in Central Jordan (56.0%), with 24.0% in North Jordan and 20.0% in South Jordan. It means that the participants of the study were represented by various geographical regions, with the central region being more represented.

The results also indicate significant trends in the social media behavior of the respondents. Most of the respondents indicated that they spent 4-6 h a day on social media (39.0%), followed by 1-3 h (30.5%), and more than 6 h (24.5%), and only 6.0% spent less than 1 h a day. This demonstrates that many of the participants were active on social media. Talking about the most popular platform, Instagram (29.5) was the most frequent, and Tik Tok (26.0) was immediately placed after it. Facebook (15.5%), Snapchat (13.5%), YouTube (7.0%), and X (6.0) were also less frequently reported.

**Figure 1:**
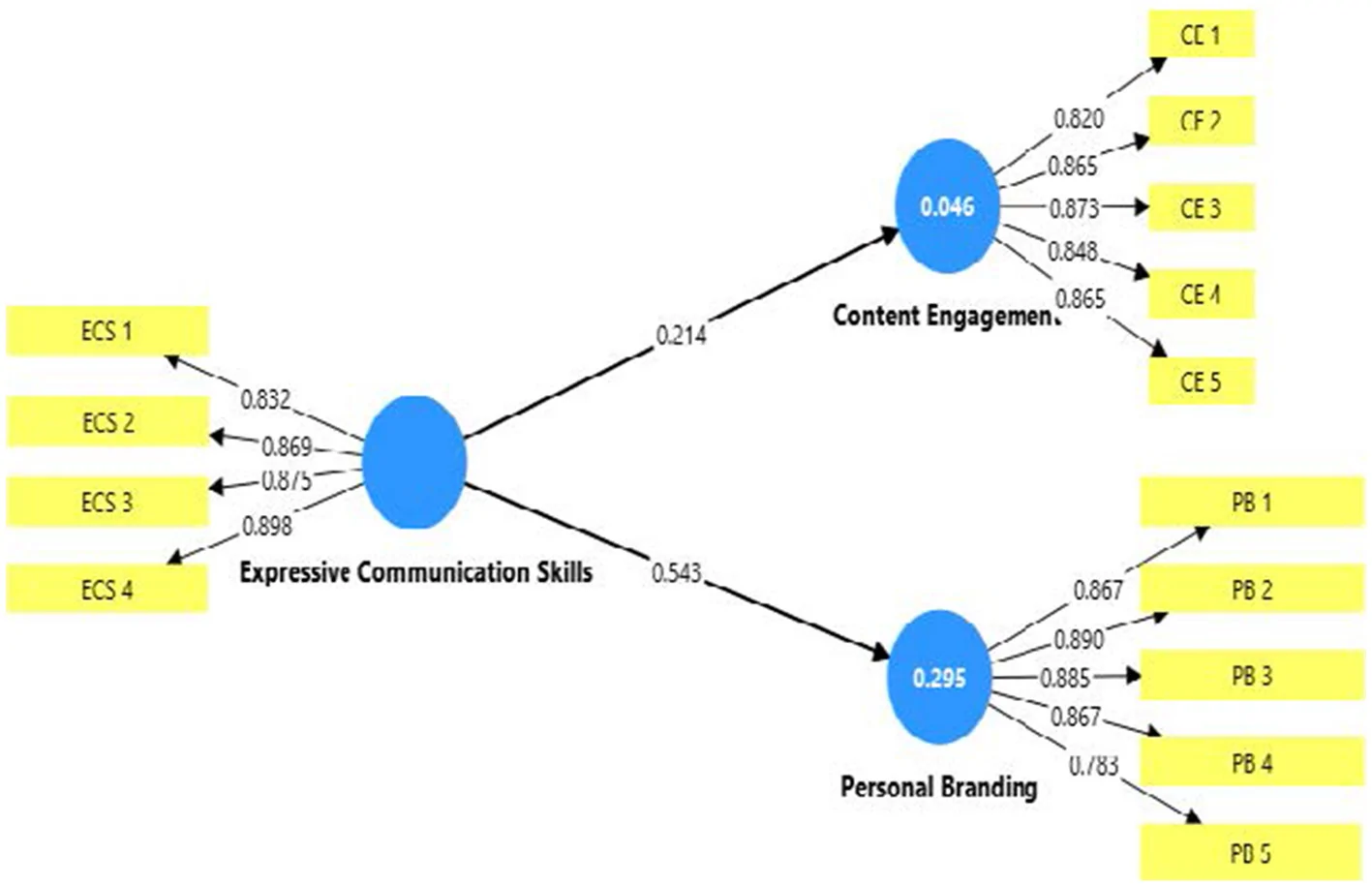
Measurement model assessment.

**Figure 2:**
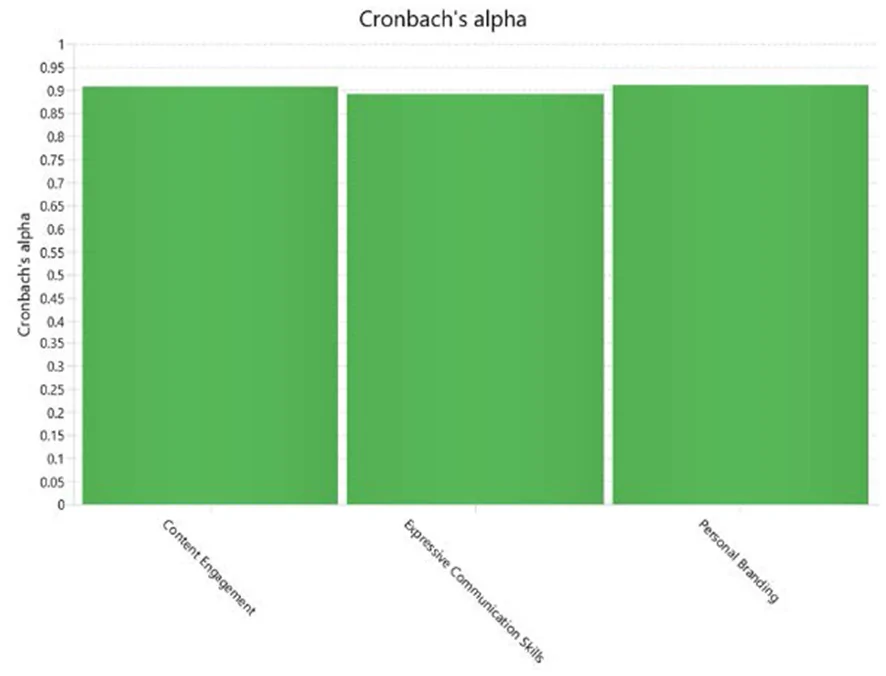
Construct reliability and validity Cronbach's Alpha.

Table 2 and Figures 1, 2 show that the measurement model achieved satisfactory levels of indicator reliability, internal consistency reliability, and convergent validity. The outer loadings ranged from 0.783 to 0.898, exceeding the acceptable threshold of 0.70 for reflective indicators (Sarstedt et al., 2021). Similarly, Cronbach's alpha values (0.892-0.911) and composite reliability values (0.925-0.934) were all above the recommended cut-off value of 0.70, indicating strong internal consistency among the measurement items (Sarstedt et al., 2021). Moreover, the AVE values (0.730-0.755) exceeded the benchmark of 0.50, confirming that the constructs explained a substantial proportion of variance in their indicators and thereby established convergent validity (Sarstedt et al., 2021). Accordingly, the findings confirm that the measurement model used in this study was reliable and valid.

**Figure 3:**
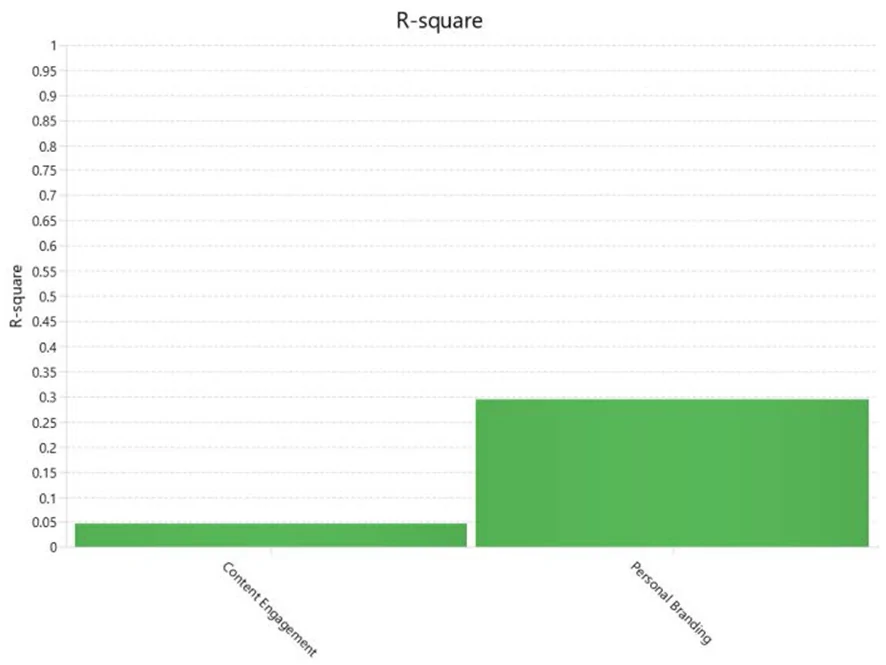
R-square.

Table 3 and Figure 3 report the coefficient of determination (*R*^2^) for the endogenous constructs in the structural model. Expressive communication skills explained 4.6% of the variance in content engagement and 29.5% of the variance in personal branding. The *R*^2^ value for content engagement is low, indicating weak explanatory power and suggesting that content engagement is influenced by several other factors beyond expressive communication skills. Therefore, this result should be interpreted cautiously. In contrast, the *R*^2^ value for personal branding indicates stronger explanatory power. In PLS-SEM, *R*^2^ is used to assess the explanatory power of the model for endogenous constructs (Sarstedt et al., 2021). Accordingly, the results suggest that expressive communication skills explain personal branding more effectively than content engagement in the present study.

**Table 3:** R-square.

Dependent variables	*R*-square	*R*-square adjusted
Content engagement	0.046	0.044
Personal branding	0.295	0.293

Table 4 presents the f-square effect size results. The *f* ^2^ value for the path from Expressive Communication Skills to Content Engagement was 0.048, indicating a small effect. This means that expressive communication skills make a limited but meaningful contribution to explaining content engagement. By contrast, the f^2^ value for the path from Expressive Communication Skills to Personal Branding was 0.418, indicating a large effect. This shows that expressive communication skills make a substantial contribution to explaining personal branding in the proposed model. Thus, the effect-size results should be interpreted according to the direction of the proposed paths: expressive communication skills influence content engagement and personal branding, not the reverse.

**Table 4:** f-square.

Variables	*f-*square
Expressive Communication Skills -> Content Engagement	0.048
Expressive Communication Skills -> Personal Branding	0.418

Table 5 shows the HTMT values for testing the discriminant validity of the study constructs. The HTMT ratios between Expressive Communication Skills and Content Engagement, Personal Branding and Content Engagement, and Personal Branding and Expressive Communication Skills were 0.236, 0.293, and 0.594 respectively. Based on the guidelines, the HTMT value must be less than 0.85 or 0.90 to establish the discriminant validity (Sarstedt et al., 2021). All reported values are below these thresholds, which means that the results indicate that the constructs are empirically distinct and that the discriminant validity is satisfactory. Thus, the measurement model exhibits good construct separation and is deemed suitable for subsequent structural model analysis.

**Table 5:** HTMT Values for Discriminant Validity Assessment.

Variables	Content engagement	Expressive communication skills	Personal branding
Content Engagement			
Expressive Communication Skills	**0.236**		
Personal Branding	0.293	**0.594**	

Bold number is threshold value.

The results of the model fit are shown in Table 6. The SRMR value for the estimated model was 0.075, which is less than the recommended value of 0.08 and represents an acceptable model fit. Moreover, the NFI value of 0.909 is greater than the widely accepted 0.90 value, which further indicates the adequacy of the model. The estimated model has higher values for d_ULS, d_G and chi-square than the saturated model, but the overall fit indices indicate that the proposed model fits the data reasonably well.

**Table 6:** SRMR.

Model Fit- SRMR	Saturated model	Estimated model
SRMR	0.052	0.075
d_ULS	0.285	0.588
d_G	0.141	0.148
Chi-square	338.572	351.087
NFI	0.912	0.909

Table 7 presents the collinearity statistics using the Variance Inflation Factor (VIF). The VIF values ranged from 1.679 to 3.537, which are all below the recommended threshold of 5.0. This indicates that there are no serious multicollinearity issues among the measurement items. Although PB 2 recorded the highest VIF value of 3.537, it remains within the acceptable range. Therefore, the collinearity results support the suitability of the items for further analysis.

**Table 7:** Collinearity Statistics (VIF).

Items	VIF
CE 1	2.192
CE 2	2.547
CE 3	2.966
CE 4	2.548
CE 5	2.575
ECS 1	2.036
ECS 2	2.453
ECS 3	2.643
ECS 4	2.879
PB 1	2.903
PB 2	3.537
PB 3	3.233
PB 4	2.830
PB 5	1.679

## Hypothesis testing and discussion

H1: Expressive Communication Skills significantly and positively influence Content Engagement among university students in Jordan.

The first hypothesis proposed that expressive communication skills significantly and positively influence content engagement among university students in Jordan. This hypothesis is supported by the results of the structural model. The path coefficient from Expressive Communication Skills to Content Engagement was positive and statistically significant (β = 0.214, *t* = 2.900, *p* = 0.004), as shown in the hypothesis testing Table 8, which means that the higher the expressive communication skills, the higher the content engagement. The *p*-value is lower than the commonly used significance threshold of 0.05 and the *t*-value is higher than the recommended critical value of 1.96, which means that the relationship is statistically significant (Sarstedt et al., 2021). As such, H1 was accepted.

**Table 8:** Hypotheses testing.

Hypothesis	Path	Beta (O)	Sample Mean (M)	Std. Deviation	*t*-value	*p*-value	Decision
H1	Expressive Communication Skills → Content Engagement	0.214	0.222	0.074	2.900	0.004	Supported
H2	Expressive Communication Skills → Personal Branding	0.543	0.544	0.045	11.962	0.000	Supported

O, original sample; M, sample mean; Std. Deviation, standard deviation; *t*-value, test statistic.

The positive coefficient of H1 is in line with the literature. Students who can express ideas, emotions and messages in an interesting way might be more likely to elicit audience reactions. When it comes to social media, engagement involves viewing, liking, commenting and sharing, and how messages are delivered can make a difference in whether they're seen and acted upon. This interpretation aligns with the research that indicates that communicative style, multimodal expression, authenticity, visual storytelling, and interpersonal meaning can foster online interaction (Alshehri and Imran, 2025; Kang et al., 2023; Munaro et al., 2024; Nurrachmah, 2025; Xia and Hafner, 2021).

The R^2^ for content engagement (0.046) is also low, so the path significance should be interpreted with caution. Expressive communication skills explain just 4.6% of the variance and are a statistically significant but weak stand-alone predictor of engagement. This trend is reasonable, as engagement is a situational response that depends on the communicator's overall performance and the quality, relevance, novelty, emotional and visual appeal of a specific post, the visibility of a post in the algorithmic recommendation system, the frequency and timing of posting, the credibility of the communicator and their previous relationship with the audience, and the characteristics of the audience, such as their interests, motivations, demographics, and platform-use habits (Barger et al., 2016; Galdón-Salvador et al., 2024; Kang et al., 2023; Lee, 2019; Munaro et al., 2024). The relatively narrow model, with one predictor only, was developed to focus on the role of expressive communication and not to maximize prediction. Further, the content-engagement items are more specific to responses to brand-generated content by influencers, while the expressive communication skills were assessed more generally as a personal ability; this may have reduced the explained variance. H1 therefore does not suggest that expressive communication is the primary factor of engagement, but rather that there is a relationship. Content quality, platform exposure, audience characteristics, source credibility, posting frequency and timing, and interaction effects across platforms should be included in future models.

H2: Expressive Communication Skills significantly and positively influence Personal Branding among university students in Jordan.

The second hypothesis was that expressive communication skills have a significant and positive effect on personal branding among the university students in Jordan. This hypothesis is supported by the results of the structural model. The path coefficient of Expressive Communication Skills to Personal Branding was positive and significant (β = 0.543, *t* = 11.962, *p* < 0.001), meaning that students with higher scores on Expressive Communication Skills are more likely to have higher scores on Personal Branding. The *p*-value is less than 0.05 and the *t*-value is much greater than the critical value of 1.96, which means that the relationship is statistically significant (Sarstedt et al., 2021). H2 was supported.

In addition to the statistical significance, the explanatory contribution to personal branding was more pronounced than to content engagement, for expressive communication skills. The *R*^2^ value of Personal Branding was 0.295, which means expressive communication skills explained 29.5% of the variance in Personal Branding. For PLS-SEM, *R*^2^ represents the amount of variance explained by the structural model for the endogenous constructs (Sarstedt et al., 2021). This finding indicates a moderate level of explanatory power and validates that the ability to express oneself in communication is a key aspect in the development and maintenance of the personal brand in digital environments.

The closer relationship with personal branding is conceptually in line with the Self-Presentation Theory. Personal branding relies on the uniformity of the communication of identity, values, competency and personality. Clear and confident expression puts students in a better position to develop a coherent and recognizable online presence. Expressive communication is not just about visibility; it's about how a user can convey a unique value proposition and maintain authenticity in their posts and interactions (Amer, 2024; Mitrić, 2022; Tewatia and Majumdar, 2022).

This discovery also takes digital identity research to a context-sensitive level. Labrecque et al. (2011) states that personal branding is a strategic communication that is designed to influence the perception of others. Based on the current evidence, it can be concluded that this process is applicable to Jordanian university students, as social media is a significant space for self-expression, identity construction, and social validation (Abdallah et al., 2024; Migdadi and Abukhadair, 2025). In a context where the community has expectations and where Arabic–English communication practices are prevalent, expressive skills can be used to balance authenticity, social acceptability and professional visibility. The study thus shows that the explanatory power of the self-presentation and digital identity perspectives can be applied to an under-researched Arab context and is culturally and linguistically sensitive.

This interpretation is also confirmed by the results of the effect sizes. The f^2^ value of the path from Expressive Communication Skills to Personal Branding was greater than the f^2^ value of the path from Expressive Communication Skills to Content Engagement. This means that expressive communication skills are more closely connected with self-presentation and identity construction than with audience engagement behaviors. Put another way, while communication skills can facilitate interaction, they seem to be even more important in enabling students to express and present themselves online.

In general, H2 is supported by strong empirical evidence that expressive communication skills are predictors of personal branding. Results indicate that expressive communicators are more successful in developing a clear, authentic and recognizable social media presence. The implications of this contrast (between the size of the impact on personal branding and on content engagement) are theoretically significant: expressive skills seem to be more important for identity construction than for audience engagement in the short term.

## Theoretical contributions, practical implications, limitations, and future research

Study contributes in three ways to the theory. First, it expands the Self-Presentation Theory by operationalizing the expressive communication skills as an antecedent of digital impression management instead of only describing the self-presentation as a performance. The impact on personal branding demonstrates the importance of verbal, emotional and relational skills for the conscious development of a recognizable online identity. Second, the study brings together self-presentation and relational communication perspectives in the field of digital identity research by looking at two different outcomes of expression in the same model: audience response (content engagement) and identity development (personal branding). The results for personal branding are significantly higher than for engagement behavior, suggesting that the explanatory and effect-size results are more closely related to identity construction than to immediate engagement behavior. Third, the study provides context-specific evidence from Jordan, an underrepresented Arab context where the norms of the community, the bilingual Arabic-English use, and social expectations may impact online identity management. The contribution is thus not in the form of a new theory but an empirical and contextual extension which makes it clear where and for which outcome existing theories have greater explanatory value.

The results are subject to several limitations. First, cross-sectional design does not allow causal conclusions to be drawn but only associations. Second, convenience sampling introduces the possibility of self-selection and coverage bias because of the accessibility, willingness, internet access, and exposure to the online survey. The sample could overrepresent digitally active students and students from Central Jordan and should not be assumed to be statistically representative of all Jordanian university students. Therefore, caution should be used in generalizing to less active users, non-student populations, other age groups, or other cultural settings. Future research should consider stratified or probability sampling according to university type, region, gender and study level, and should attempt to compare the respondents with the nonrespondents, if possible. Third, recall and social-desirability bias might affect self-reported measures. Lastly, the low R^2^ for content engagement indicates that key factors were not included. Content quality and relevance, visual and emotional appeal, platform algorithms and affordances, audience characteristics, source credibility, posting frequency and timing, and peer influence should be tested in future research. Longitudinal, experimental and cross-platform designs would yield better evidence concerning the temporal order and context-specific effects.

The practical implications are not limited to the training of students. Digital self-presentation, awareness of audience, storytelling, clarity of the message, and ethical internet identity management can all be part of the communication, career-development and employability curriculum. These findings can be used to assist students in creating coherent professional profiles and articulating their skills and accomplishments in an authentic way, by career centers. Digital literacy programs should also educate students about the algorithms of platforms, the quality of content, posting patterns, and audience demographics, so that they don't think that communication skill alone will ensure interaction. The findings are useful for training institutional communication teams and professional practitioners in the design of authentic messages, adaptation to other languages and modes, segmentation of audiences, and planning of content for different platforms. Expressive skills are particularly important for credibility and personal-brand consistency, and when paired with quality content, posting frequency and timing, and evidence-based audience analysis, can lead to increased engagement for communication practitioners and content creators. Since the impact on content engagement was statistically significant but small, interventions should be assessed through behavioral measures in addition to perceived communication competence.

## Conclusion

This paper examined how expressive communication skills influence content engagement and personal branding among university students in Jordan. The results showed that expressive communication skills had significant positive effects on both outcomes. However, the explanatory power for content engagement was relatively low, indicating that expressive communication skills explain only a limited proportion of audience interaction. In contrast, the effect on personal branding was stronger, suggesting that expressive communication skills are especially important for helping students present themselves clearly, authentically, and consistently on social media.

Overall, the study highlights the growing importance of expressive communication skills in Jordan's digital environment, where social media platforms have become central spaces for interaction, self-expression, and identity construction. The findings suggest that improving expressive communication skills may help university students strengthen both their online engagement and personal branding efforts. At the same time, the limited R^2^ value for content engagement indicates that future studies should examine additional factors that shape digital engagement and online self-presentation.

## Data Availability

The raw data supporting the conclusions of this article will be made available by the authors, without undue reservation.
